# Repeated bolus injections of bupivacaine for continuous bilateral transversus thoracis plane block undergoing median sternotomy in a dog: A case report

**DOI:** 10.17221/118/2023-VETMED

**Published:** 2024-04-25

**Authors:** Dalhae Kim, Dongmin Shin, Sookyung Yun, Gayeon An, Joohyun Jung, Won-gyun Son

**Affiliations:** ^1^Ilsan Animal Medical Center, Goyang, Republic of Korea; ^2^Department of Veterinary Clinical Sciences, College of Veterinary Medicine, Seoul National University, Seoul, Republic of Korea

**Keywords:** acute pain, indwelling catheter, lobectomy, thoracotomy, ultrasound-guided

## Abstract

An 8-year-old, 6.5 kg, neutered female Shih-Tzu dog was presented for surgical resection of a mediastinal mass. A median sternotomy and left cranial lung lobectomy were performed. Intraoperatively, with the patient under general anaesthesia, a bilateral transversus thoracis plane (TTP) block was performed by injecting 0.5% bupivacaine (0.2 ml/kg) per side using real-time ultrasound guidance. After surgery, indwelling catheters for repeated bolus injections of bupivacaine in TTP were placed as follows: the fifth sternebra was palpated in dorsal recumbency, and the transducer was placed in the longitudinal plane lateral to the sternal border. A 16 gauge over-the-needle catheter was inserted caudo-cranially using an in-plane technique and located in the TTP. An intermittent bolus of bupivacaine (0.1 ml/kg) per side was injected via the indwelling catheter every 8 h for 3 days, with a constant rate infusion of an intravenous fentanyl (1 μg/kg/h) and ketamine (0.12 mg/kg/h) combination. Post-operative pain was evaluated using the Glasgow composite measure pain scale and the score was 4–5/24 on the day of surgery and gradually decreased over time. Additional rescue analgesia was not required. Repeated boluses of bupivacaine for a continuous bilateral TTP block may be a useful adjuvant for perioperative pain management strategies, including median sternotomy, in dogs.

A median sternotomy is an invasive surgical procedure that allows access to the thoracic cavity and is associated with considerable post-operative pain, leading to hypoventilation, hypoxaemia, increased morbidity, delayed recovery, and prolonged hospitalisation (Dhokarikar et al. 1996; Abd El-Hamid and Azab 2016; Perez et al. 2023). Sensory innervation of the sternum is facilitated by the intercostal nerves and the ventral branches of the thoracic nerves. These nerves also provide motor function to the intercostal muscles and sensory innervation to the muscles, parietal pleura, and overlying skin (Dhokarikar et al. 1996; Mazzeffi and Khelemsky 2011; Evans and Lahunta 2013; Perez et al. 2023). In order to achieve adequate analgesia during sternotomy, it is essential to anaesthetise the 2^nd^ to 7^th^ thoracic intercostal nerves bilaterally (Mazzeffi and Khelemsky 2011; Evans and Lahunta 2013). Opioids are still considered the standard systemic analgesic for thoracic surgery; however, their use is associated with reduced pulmonary residual capacity, respiratory depression, and hypoxaemia (Dhokarikar et al. 1996). Therefore, the use of locoregional anaesthesia in thoracic surgery is recommended for a balanced anaesthesia approach.

The transversus thoracis plane (TTP) block is a technique used in human medicine for the primary purpose of providing perioperative analgesia to patients undergoing a median sternotomy, chest tube placement, and cardiac surgery (Aydin et al. 2020; Cakmak and Isik 2021). Recent studies by Alaman et al. (2021) and Zublena et al. (2021) described two ultrasound (US)-guided approaches for TTP block in dog cadavers, which were performed by injecting a local anaesthetic solution into the interfascial plane between the transversus thoracis muscle and the internal intercostal muscles. The TTP block could be useful for analgesia of the ventral chest wall, especially in a median sternotomy, numbing several intercostal nerves with a single injection site (Alaman et al. 2021; Zublena et al. 2021). In addition, when performed bilaterally, the US-guided TTP block may be an appropriate alternative to previously reported techniques such as thoracic epidural anaesthesia, thoracic paravertebral block, serratus plane block, erector spinae plane block, and intercostal blocks (Flecknell et al. 1991; Thompson and Johnson 1991; Pascoe and Dyson 1993; Portela et al. 2017; Portela et al. 2019; Asorey et al. 2021).

Barrientos and Merlin (2022) described the clinical application of a TTP block in dogs, and Perez et al. (2023) described it in cats; however, the continuous TTP block technique has not been described in veterinary medicine. This case report describes the use of a repeated bolus injection of bupivacaine for a continuous bilateral TTP block as part of a multimodal analgesia protocol to improve pain management in a dog that underwent a median sternotomy.

## Case report

An 8-year-old, 6.5-kg, spayed female Shih-Tzu was referred to the animal medical centre for surgical resection of a mediastinal mass, which had been identified during a health check-up at the referring hospital. A complete blood count analysis yielded unremarkable findings except for an increased white blood cell count (51.8 × 10^9^/l, reference range: 5.05–16.8), and a mildly decreased haematocrit level (36.4%, reference range: 37.3–61.7%). The serum biochemical analysis yielded unremarkable findings except for increased alkaline phosphatase levels (36.5 μkat/l, reference range: 0–1). The electrolyte analysis, urinalysis, and electrocardiography did not reveal any significant findings. The thoracic radiography revealed a large mediastinal mass. Computed tomography (CT) was performed, and the images showed a large, predominantly soft tissue attenuating mediastinal mass filling the cranioventral thoracic cavity. The mass was 5.5 cm in length, 5.3 cm in height, and 6.6 cm in width. The mass was mildly enhanced following contrast administration and had multifocal, non-enhancing, coalescent hypoattenuating regions (8–45 HU). Although the cranial *vena cava* was dorsally displaced and compressed by the mass, there was no evidence of vascular invasion. Adhesion to the left cranial lung lobe was suspected due to the irregular boundary between the mass and the lung lobe. Pleural effusion was not observed. After a fine-needle aspiration examination, thymoma was first considered, and a median sternotomy was scheduled 8 days after the CT.

On the day of the procedure, food was withheld for 8 h and water was withheld for 2 h prior to anaesthesia. The cephalic vein was catheterised using a 24-gauge over-the-needle polyurethane catheter, and Hartmann’s solution (Dai Han Pharm., Ansan, Republic of Korea) was administered intravenously at an initial rate of 5 ml/kg/h. The dog was pre-treated with intravenous maropitant (1 mg/kg; Cerenia^®^; Zoetis, Seoul, Republic of Korea), cefazolin (22 mg/kg; Chong Kun Dang Corp., Republic of Korea), midazolam (0.2 mg/kg; Midazolam; Bukwang Pharmaceuticals, Seoul, Republic of Korea), hydromorphone (0.05 mg/kg; Hana Pharmaceuticals, Hwaseong, Republic of Korea), and ketamine (0.1 mg/kg; Yuhan Pharmaceutical, Seoul, Republic of Korea) following pre-oxygenation with 100% flow-by oxygen. Thereafter, a constant rate infusion (CRI) of ketamine (0.6 mg/kg/h) was initiated. General anaesthesia was induced with propofol (4 mg/kg; Daewon Pharm., Seoul, Republic of Korea) and the animal was orotracheally intubated. Anaesthesia was maintained with isoflurane (1.0–1.3%; I-Fran Liquid; Hana Pharmaceuticals, Hwaseong, Republic of Korea) in 100% oxygen after intubation. The heart rate and rhythm, end-tidal partial pressure of carbon dioxide (EtCO_2_), oesophageal temperature, pulse oximetry, and end-tidal isoflurane concentration were continuously monitored using a multi-parameter monitor (B125 patient monitor; GE Healthcare, Chicago, USA). The blood pressure was measured using an oscillometric blood pressure monitoring device (Vet25; SunTech Medical, Inc., Morrisville, NC, USA). All the data were recorded at 5-min intervals.

With the dog in dorsal recumbency, the surgical site was prepared, and a 1–15 MHz linear transducer (Aloka Alpha 7; Hitachi Healthcare, Twinsburg, USA) was positioned lateral to the sternum and parallel to it (parasagittal plane) on the ventral aspect of the chest, in a parasternal orientation lateral to the fifth and sixth costal cartilages. The target image consisted of an intercostal space in the centre of the screen, with two ribs delimiting it on both sides. The pectoral, internal intercostal, and transversus thoracis muscles were identified from superficial to deep. An 88 mm 22-gauge needle (Spinocan^®^; B. Braun, Melsungen, Germany) was then inserted caudo-cranially with an in-plane technique. The needle tip was positioned in the TTP fascial plane between the transverse thoracis and the internal intercostal muscles. A small amount of 0.9% normal saline was injected to confirm the presence of the needle tip in the target plane by observing the hydrodissection of the fascial plane. Bupivacaine hydrochloride 0.5% (0.2 ml/kg; Myungmoon Pharmaceutical, Seoul, Republic of Korea) was then administered. The same procedure was performed for the left hemithorax (total injected volume, 0.4 ml/kg). The TTP block was performed over a period of 5 minutes.

The patient was transferred to the operation theatre and was positioned in dorsal recumbency. At this stage, the patient was connected to a rebreathing circuit system (NXT 9100c; GE Healthcare, Chicago, USA). Mechanical ventilation was applied to maintain an EtCO_2_ of 35–45 mmHg with a peak inspiratory pressure of 9–10 cmH_2_O. There were periods of hypotension (defined as a mean arterial pressure of < 60 mmHg) during general anaesthesia. Three i.v. ephedrine boluses (0.05 mg/kg; Jeil Pharm., Seoul, Republic of Korea) and a CRI of dobutamine (5 μg/kg/min; Myungmoon Pharm., Seoul, Repubic of Korea) were required to treat the hypotension.

A median sternotomy was performed. The mediastinal attachments were dissociated using monopolar electrocautery with a LigaSure vascular sealing device. A mass filling the chest cavity was identified in front of the heart. There was considerable adhesion between the mass and left cranial lobe of the lung; therefore, a lung lobectomy was performed using a stapler (Covidien Endo GIATM reinforced reload with tri-staple technology; Medtronic, Minneapolis, USA). The phrenic nerve passed through the mass, and after en-bloc resection, the mass was extracted.

After confirming that the aorta, vagus nerve, and caudal *vena cava* were intact, a submerged breath-hold was performed without evidence of leakage from the pulmonary parenchyma. A Barovac drain tube was placed, and the median sternotomy was routinely closed.

**Figure 1:**
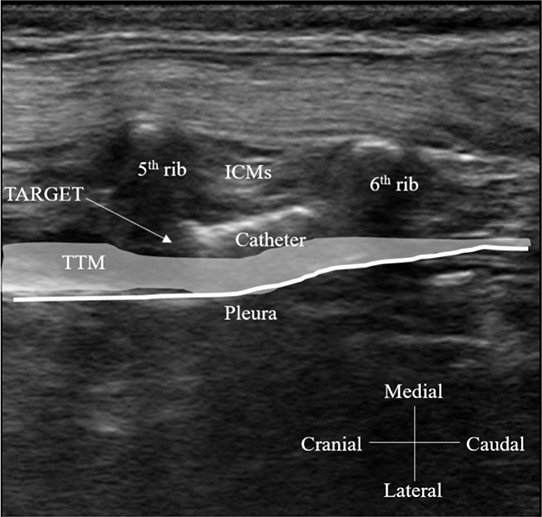
Representation of the sonoanatomy of the transversus thoracis plane block between the 5^th^ and 6^th^ costal cartilages in a dog, with the catheter advanced at the injection site. The catheter and costal pleura (solid white line) are visible ICMs = intercostal muscles; TTM = transversus thoracis muscle

After the closure, indwelling catheters for the repeated bolus of bupivacaine injections in the TTP were placed. The catheter placement was performed using real-time US with a high-frequency linear transducer. The dog was positioned in dorsal recumbency, and the transducer was positioned lateral to the sternum and parallel to it (parasagittal plane) on the ventral aspect of the chest, in a parasternal orientation lateral to the fifth and sixth costal cartilages. This time, a 14-gauge, 13 cm over-the-needle catheter (Milacath^®^; Mila International Inc., Florence, USA) was inserted caudo-cranially with an in-plane technique. The needle tip was positioned in the TTP fascial plane between the transversus thoracis and the internal intercostal muscles (Figure 1).

**Figure 2:**
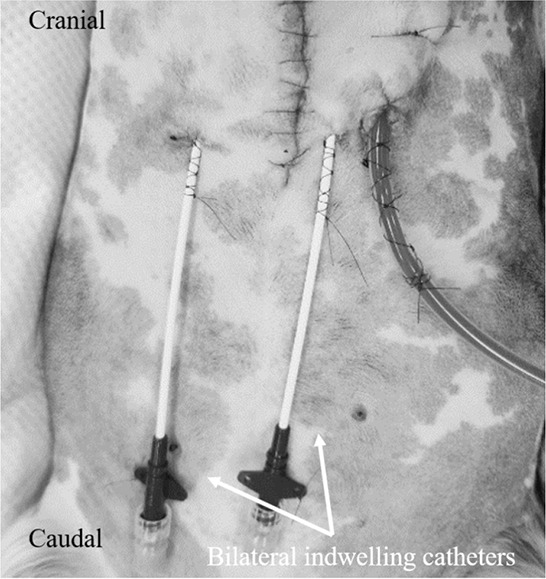
A dog with bilateral indwelling catheters (14-gauge over-the-needle catheter) to provide the repeated bolus injection of bupivacaine for the continuous transversus thoracis plane block

The same procedure was followed for the contralateral hemithorax and the catheters were fixed using a Chinese finger knot. After the needle was removed, an intravenous cap (male Luer lock injection cap; Korea Vaccine, Ansan, Republic of Korea) was installed (Figure 2).

To prevent infection, the externally exposed catheter and i.v. cap were sealed with a transparent film (Tegaderm^TM^ Film; 3M Health Care, Minnesota, USA). The placement of the bilateral catheters for the TTP block took 15 minutes.

The duration of the surgery was 140 min, and the duration of the anaesthesia was 180 minutes. Thermal support was provided through a Darvall Cozy Warming Blanket System and a hot pack application. The patient was extubated 15 min after the discontinuation of isoflurane.

The dog was admitted to the intensive care unit. The post-operative multimodal analgesia consisted of an intravenous CRI of fentanyl (1 μg/kg/h) and ketamine (0.12 mg/kg/h) combination for 2 days combined with an intermittent bolus of 0.5% bupivacaine (0.1 ml/kg each) injected via an indwelling catheter bilaterally (total 0.2 ml/kg) every 8 h for 3 days. Normal (0.9%) saline (0.1 ml/kg each) was injected to flush the local anaesthetic agent via the indwelling catheters. The correct placement of the catheters was confirmed through a thorax X-ray taken every morning. The post-operative pain was evaluated using the Glasgow Composite Measure Pain Scale Short Form (CMPS-SF) (Reid et al. 2007) every 2 h throughout the day and then every 8 h by trained veterinary nurses after anaesthesia (Table 1). The CMPS-SF score was 4–5/24 on the day of surgery and the values of the CMPS-SF gradually decreased over time. The catheters were removed on the third post-operative day.

**Table 1:** Pain scoring using the Glasgow composite measure pain scale-short form every 2 h on the day of surgery and every 8 h thereafter. 0 h is the time when indwelling catheters were installed

	2 h	4 h	6 h	8 h	10 h	12 h	20 h	28 h	36 h	44 h	52 h	60 h
Pain score	5	5	5	4	4	4	3	2	2	1	1	0

The dog was discharged from the hospital under the care of its owner four days after the surgery. The histopathological examination of the cranial mediastinal mass revealed a thymoma.

## DISCUSSION

Thoracic surgery is more painful than any other surgery, and the pain associated with the surgery is difficult to control (Dhokarikar et al. 1996; Abd El-Hamid and Azab 2016; Perez et al. 2023). Patients who have undergone thoracic surgery must continue to use damaged chest muscles to breathe; however, breathing through the damaged muscles does not let them breathe deeply and makes them more susceptible to hypoventilation and hypoxaemia if the pain is not well-controlled after thoracic surgery (Dhokarikar et al. 1996; Abd El-Hamid and Azab 2016; Perez et al. 2023). In addition, acute post-operative pain after a median sternotomy, if not properly managed, can lead to pulmonary complications, haemodynamic consequences, prolonged hospitalisation, and chronic neuropathic pain (Dhokarikar et al. 1996; Abd El-Hamid and Azab 2016; Perez et al. 2023). In order to achieve adequate analgesia during the sternotomy, local anaesthesia is essential and this is the first case report describing a repeated bolus injection of bupivacaine for a continuous bilateral TTP block after a median sternotomy in a dog.

One of the major consequences of thoracic surgery on the lung function is a decrease in the functional residual capacity, which may lead to alveolar collapse. In addition, the administration of opioids, such as fentanyl, after i.v. boluses or CRI has been associated with respiratory distress in human and veterinary patients (Baxter et al. 1994). In dogs, the systemic administration of opioids after thoracotomy results in severe ventilatory depression, whereas ventilatory parameters are not significantly affected after an intercostal nerve block (Thompson and Johnson 1991). Similarly, compared to systemic morphine, interpleural bupivacaine reduces the respiratory effort and improves the gas exchange after lateral thoracotomy (Conzemius et al. 1994; Stobie et al. 1995). These findings suggest the superiority of locoregional analgesia over systemic post-operative analgesic techniques and support the absence of ventilatory dysfunction secondary to the intercostal nerve block (Thompson and Johnson 1991; Conzemius et al. 1994; Zublena et al. 2021). Therefore, multimodal analgesic strategies including locoregional analgesia are important.

In human medicine, the US-guided TTP block is a locoregional analgesic technique that is considered effective and safe with a high success rate, reducing the need for systemic opioids to provide pain relief in patients undergoing a median sternotomy (Fujii et al. 2019; Kar and Ramachandran 2020; Kumari et al. 2022; Perez et al. 2023). Previous case reports have described both a single injection and continuous catheter infusions for the relief of sternotomy pain, which are effective in human medicine (Fujii et al. 2019; Kumari et al. 2022). In veterinary medicine, Barrientos and Merlin (2022) reported on a dog that underwent a median sternotomy and lobectomy, and Perez et al. (2023) reported the clinical use of this block in a cat undergoing a median sternotomy; these case reports used single injections and required rescue analgesia 7 to 8 h after the TTP block. To provide long duration of locoregional analgesia after surgery, an indwelling catheter should be installed owing to the limited duration of action of local anaesthetics.

The placement of indwelling catheters allows the repeated administration of local anaesthetics. Multi-hole catheters or adapted epidural catheters have been used to provide continuous nerve blocks, which not only improve pain relief, but also reduce the need for opioid analgesics and allow patients to be discharged sooner (Armitage-Chan 2013). However, in this case, an over-the-needle catheter could only be used in this hospital, in which the hole was only present at the catheter tip. The exact anatomical location was determined using US guidance.

A previous cadaveric study conducted by Zublena et al. (2021) described the sagittal approach to the TTP block; however, the study involved three injections (third, fifth, and seventh intercostal spaces). Another cadaveric study conducted by Alaman et al. (2021) described a transverse approach to the TTP block involving a single injection between the fifth and sixth costal cartilages. Veterinary case reports have applied the transverse approach involving a single injection; however, the sagittal approach has been judged to be more helpful for catheter placement and maintenance. Therefore, the sagittal approach was also applied with the catheter tip placed between the fifth and sixth costal cartilages. The decision to modify the approach was based on a case report of a single injection using the sagittal approach in human medicine (Kumari et al. 2022).

When administered alone, bupivacaine typically has an onset time of 15 min and a duration of effect of 6–8 h (Almasi et al. 2020), and 1–1.5 mg/kg of 0.5% bupivacaine can be gently infused every 6–8 hours. In this case, an intermittent bolus of 0.5% bupivacaine (0.5 mg/kg = 0.1 ml/kg each) was injected via the indwelling catheter bilaterally (total 0.2 ml/kg) every 8 h for 3 days. Normal saline (0.9%) was used to push the local anaesthetic into the catheter at 0.1 ml/kg.

Using the TTP block as part of the anaesthetic protocol instead of other locoregional analgesia protocols, such as intercostal nerve blocks or thoracic epidural anaesthesia, may reduce the risk of respiratory depression because it does not affect the innervation of the intercostal muscles (Cakmak and Isik 2021). Additionally, as a wide range of nerve blocks can be administered with a single injection, the possibility of vascular or pleural puncture or pneumothorax is reduced (Alaman et al. 2021). The authors believe that the addition of the TTP block as a part of the multimodal analgesic protocol reduced the opioid consumption during the post-operative period, which may have helped in the maintenance of better pulmonary function and improved recovery quality based on the CMPS-SF observed in the present case. Possible complications described in humans, including local anaesthetic toxicity, infection, vascular or pleural puncture, haematoma, pneumothorax, and anaphylactic shock, were not identified in this case (Baxter et al. 1994; Alaman et al. 2021).

The limitation in this case was the i.v. catheters, which only ended up in the fifth intercostal space. In the cadaver study, when the TTP block was performed in the 5^th^ intercostal space, intercostal nerves from T2 to T7 were stained, but not all the nerves were uniformly blocked (Alaman et al. 2021). Therefore, in this case as well, it is possible that the effect of the bupivacaine administration was limited to a small area around the catheter tip rather than the entire area where sternotomy was performed. In addition, the effectiveness of the TTP blockade may be masked by sufficient post-operative analgesia with systemically administered analgesics. Another limitation is the repeated bolus injection of bupivacaine for the continuous TTP block was performed in only one case. In only one case, it is impossible to confirm the efficacy/safety ratio, since different patients can respond differently and some possible complications can show up. Nonetheless, the TTP block combined with fentanyl and ketamine infusions resulted in post-operative cardiopulmonary stability, low post-operative pain scores, and subjective animal comfort in this case. In our experience, median sternotomies require the administration of high-dose post-operative analgesics to provide appropriate anti-nociception and cardiovascular stability.

In conclusion, it could be stated that in the present case, a satisfactory level of analgesia was achieved with the addition of a continuous bilateral US-guided TTP block with a repeated bolus injection of bupivacaine in a dog undergoing a median sternotomy, suggesting that the TTP block may be a useful adjuvant for perioperative pain management strategies, including a median sternotomy in dogs.
